# Enhanced antifungal and cytotoxic potential of essential oils encapsulated in polydopamine nanocapsules against *Candida albicans* and *Pichia kudriavzevii*

**DOI:** 10.1038/s41598-026-40233-y

**Published:** 2026-03-11

**Authors:** El-Sayed M. El-Morsy, Marwa T. Mohesien, Mohamed Alghzaly Mohamed Abdellatif, Elsayed Elbayoumy

**Affiliations:** 1 Microbiology and Botany Department, Faculty of Science, Damietta University, New Damietta, 34517 Egypt; 2 Chemistry Department, Faculty of Science, Damietta University, New Damietta, 34517 Egypt

**Keywords:** Essential oils, Polydopamine nanocapsules, Candida albicans, Pichia kudriavzevii, Antifungal activity, Synergistic effect, Drug delivery, Fungi, Cancer therapy

## Abstract

Oral fungal infections, particularly those caused by *Candida albicans* and *Pichia kudriavzevii*, pose significant health challenges, especially in immunocompromised individuals. This study systematically investigates the antifungal efficacy, antioxidant, and antiproliferative of essential oils (EOs) from *Mentha longifolia* and *Cupressus macrocarpa*, encapsulated in polydopamine (PDA) nanocapsules to enhance their stability and bioactivity. Compositional analysis using gas chromatography-mass spectrometry (GC-MS) identified key bioactive compounds contributing to their antifungal and antioxidant properties. The nanocapsules were further characterized using Fourier-transform infrared spectroscopy (FTIR), Transmission electron microscopy (TEM), dynamic light scattering (DLS), and zeta potential analysis, confirming their successful encapsulation, nanoscale morphology, and surface charge stability. The encapsulated EOs demonstrated significantly enhanced antifungal activity, with lower minimum inhibitory concentrations (MICs) than their non-encapsulated counterparts. Additionally, they exhibited strong antioxidant potential in the DPPH assay and notable cytotoxic effects against MCF-7 and HepG-2 cancer cell lines. TEM provided direct evidence of fungal cell disruption, including chromatin condensation, membrane damage, and cytoplasmic leakage, indicating apoptosis and necrosis. The synergistic interaction between EOs and PDA, confirmed by fractional inhibitory concentration index (FICI) analysis, highlights their potential as effective antifungal agents. These findings strongly support EO-PDA nanocapsules as a promising alternative for treating oral fungal infections while also exhibiting potential anticancer properties.

**Supplementary Information:**

The online version contains supplementary material available at 10.1038/s41598-026-40233-y.

## Introduction

The oral cavity provides a highly favorable environment for microbial colonization and growth. It harbors one of the most complex microbiomes in the human body, second only to the colon, with greater diversity than the skin and other mucosal surfaces^1–3^. To date, approximately 800 microbial species have been identified in the oral cavity, including bacteria as the predominant group, alongside fungi, archaea, protozoa, and bacteriophages^1–7^. Notably, over 100 fungal species have been recorded in the oral microbiota, many of which remain unculturable^6^. Fungal presence in the oral cavity has been recognized for centuries; Hippocrates (c. 460–370 BCE) documented cases of oral ulcers (aphthae), which are now believed to have been oral candidiasis. The condition gained significant attention during the AIDS epidemic due to its strong association with immunosuppression^8^.

*Candida* species are the most common and extensively studied fungal colonizers of the gastrointestinal tract, particularly in the oral cavity^9–15^. Oral candidiasis is not only linked to HIV infection but also to other immunocompromising conditions such as leukemia, oncologic diseases, and the effects of chemotherapy, radiotherapy, and other oral pathologies^16,17^. Beyond *Candida albicans*, other *Candida* species, including *C. glabrata*, *C. krusei* (*P. kudriavzevii*), *C. tropicalis*, and *C. parapsilosis*, have increasingly contributed to infections, particularly among immunocompromised individuals^18^.

Essential oils (EOs) are complex natural mixtures rich in bioactive compounds, primarily monoterpenes, known for their potent antimicrobial properties. Examples include carvacrol and thymol (oregano), eugenol (cloves), allyl isothiocyanate (mustard), menthol (peppermint), and allicin (garlic)^19^. EOs have demonstrated strong antifungal activity against various *Candida* species, often surpassing conventional antifungal agents in efficacy^20–23^. Recent comprehensive reviews have highlighted the resurgence of essential oils in combatting Candida albicans infections due to their multimodal mechanisms of action^24^. Approximately 60 plant families are known to produce essential oils with potential antimicrobial applications^25^.

In this study, we explore the essential oils from wild mint (*Mentha longifolia* L., Lamiaceae) and Monterey cypress (*Cupressus macrocarpa* Hartweg ex Gordon, Cupressaceae), which are traditionally used as food flavoring agents and possess antimicrobial properties^26–28^. The EO from fresh *M. longifolia* leaves is rich in pulegone, an oxygenated monoterpene with antimicrobial potential^29^. In contrast, the EO from Egyptian *C. macrocarpa* branchlets contains terpinen-4-ol (23.7%), α-phellandrene (19.2%), α-citronellol (17.3%), and citronellal compounds known for their antimicrobial and antioxidant activities^30,31^. Additionally, *C. macrocarpa* EO, particularly its sabinene and terpinen-4-ol components, has demonstrated strong antifungal activity against *Trichophyton rubrum*^32^. Essential oils derived from bioactive natural products have demonstrated potent activity against multiple *Candida auris* isolates^33^.

Polydopamine (PDA), an oxidative product of dopamine and catecholamines, has emerged as a versatile bioinspired coating material. Its ability to form uniform, conformal layers on virtually any surface makes it valuable in numerous biomedical applications^34^. PDA is highly biocompatible and has been widely studied in drug delivery systems, particularly in mucosal drug administration^35,36^. Additionally, PDA-modified nanoparticles are gaining attention in antimicrobial applications due to their multifunctionality^37^. PDA coatings have been shown to significantly reduce microbial adhesion on medical devices, including catheters, enhancing their safety and effectiveness^38,39^.

**Fig. 1:**
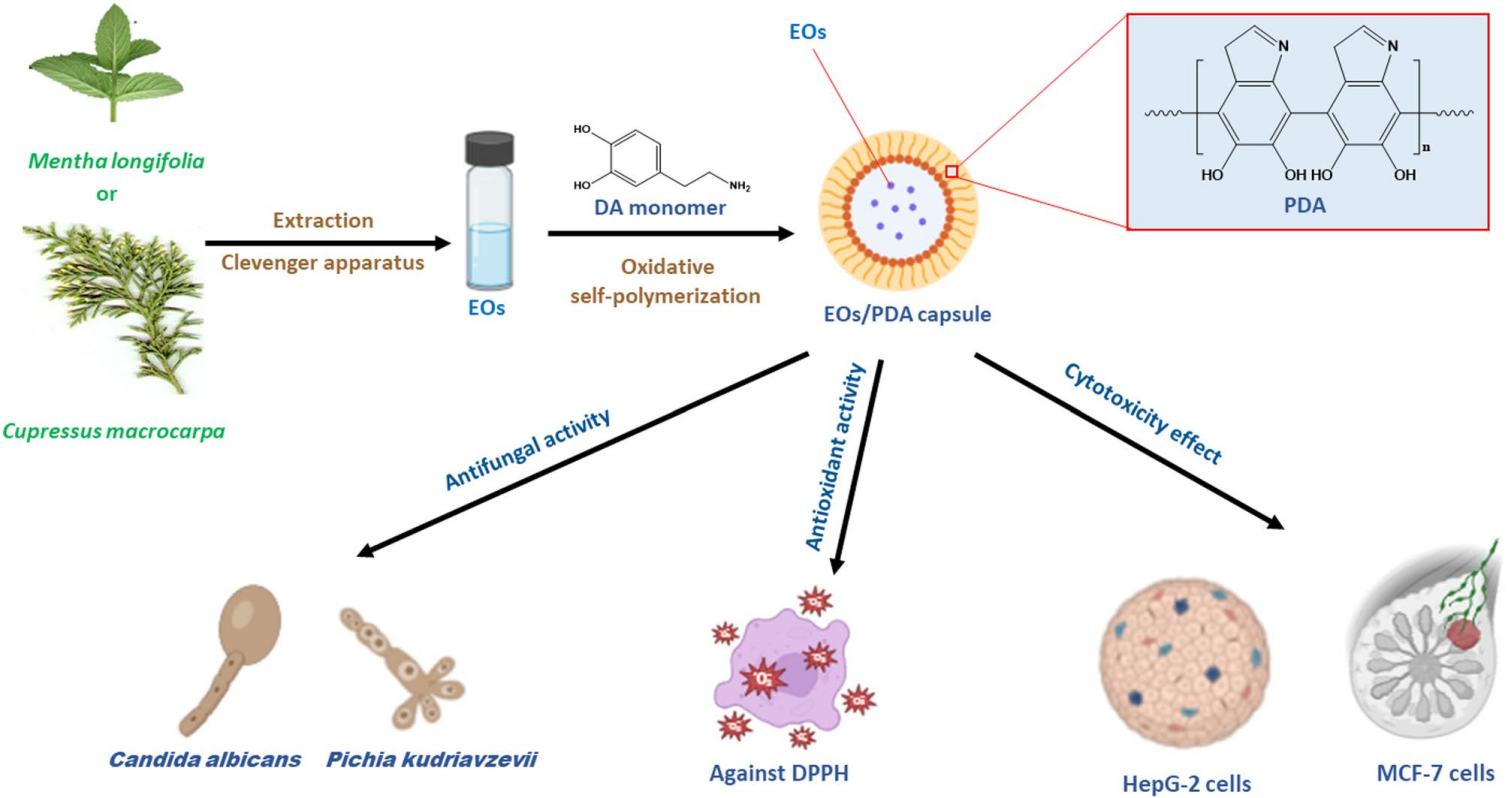
Schematic representation of the preparation and biological evaluation of essential oil (EO)-loaded polydopamine (PDA) capsules.

This study aims to investigate a novel antifungal approach by encapsulating the essential oils of *M. longifolia* and *C. macrocarpa* within PDA-based nanocarriers (Fig. 1). By leveraging the synergistic effects of EOs and PDA, we aim to develop an innovative and highly effective treatment for oral fungal infections, particularly oral candidiasis. In addition to antifungal activity, evaluating the antioxidant and antiproliferative properties of the essential oil–loaded polydopamine nanocapsules provides valuable insight into their multifunctional potential. These complementary assessments help elucidate how redox-modulating and cytotoxic characteristics may contribute to the overall antifungal mechanism by enhancing oxidative stress within fungal cells and stabilizing the bioactive components during treatment.

## Experimental techniques

### Materials

*Mentha longifolia* and *Cupressus macrocarpa* plant were purchased from Plant nursery at New Damietta city-Egypt. Dopamine hydrochloride (98%) (Sigma Aldrich), ethanol (AR, > 99.7%), ammonia (28–30%), sodium dihydrogen phosphate, disodium hydrogen phosphate, and sodium acetate were obtained from Scharlau Chemical Reagents. Acetic acid (glacial, 99%) and dimethyl sulfoxide (DMSO) were obtained from Thermo Scientific Chemical Reagents. All chemicals were used as received without any further purification.

### Plant material and preparation of essential oils

Two essential oils were extracted from the leaves of *Mentha longifolia* and *Cupressus macrocarpa* using a Clevenger apparatus, following the method outlined by Karakaya et al.^40^. In brief, the leaves of each plant species were dried. 100 g of the dried plant from each species were placed in a 500 mL Clevenger apparatus containing 300 mL of distilled water. The extraction temperature was maintained at the boiling point using a heater mantle, and the extraction process was carried out for three hours. The essential oil produced during the extraction were collected in clean bottles and stored in a refrigerator at 4 °C for further analysis.

### Synthesis of polydopamine and encapsulation of essential oils

PDA and essential oil loaded PDA nanocapsules were synthesized following the method reported by Liu et al.^41^. The synthesis was carried out in an alcohol-water mixed solvent. Ethanol (36 mL) was mixed with an aqueous ammonia solution (0.84 mL, 28–30%) and the mixture was complete to 120 mL with deionized water. The mixture was stirred magnetically at room temperature for 30 min. For the synthesis of PDA nanocapsules without essential oils, dopamine hydrochloride solution in deionized water (0.45 g, 9 mL) was then injected into the above mixed solution. As a result, the solution changed color from pale brown to dark brown. The reaction was allowed to proceed for 48 h. For the encapsulation of essential oils, the same procedure was followed in the presence of each oil (0.25 mL), which was first dispersed in ethanol prior to ammonia addition, which was first dispersed in ethanol prior to ammonia addition. The polymerization of dopamine occurred at the oil–water interface, forming a PDA shell around the oil droplets and resulting in the formation of essential oil-loaded PDA nanocapsules (EOs-PDA). After complete the reaction, PDA and EOs-PDA were collected from the reaction solution by centrifugation at 8,000 rpm for 10 min. The dark brown product was washed three times with deionized water by centrifugation at 8,000 rpm for 10 min to remove unreacted monomers and residual solvents, then dried under vacuum at room temperature and stored for further use. The symbols EO1-PDA and EO2-PDA were used to represent the encapsulated essential oils of *Cupressus macrocarpa* and *Mentha longifolia*, respectively.

### Characterization techniques

The chemical composition of the two essential oils was analyzed using a GC-TSQ mass spectrometer (Thermo Scientific 1310 gas chromatograph system, mass spectrometer MS TSQ 9000, Italy), coupled with a direct capillary TG-5MS column (30 m x 0.25 mm x 0.25 μm film thickness). Helium was used as the carrier gas, set to a column flow rate of 1.0 mL/min. Spectra were collected, and the components were identified by comparing their retention times and mass spectra to those in the WILEY 09 and NIST 14 mass spectral databases. Fourier transform infrared (FTIR) spectra were measured over a range of 4000–400 cm^− 1^ using a JASCO FT/IR-6100 spectrometer, with a KBr pellet sample. UV-visible absorption spectra were recorded at room temperature using a Jasco V-630 automatic recording spectrophotometer, with a solution in a 1 cm quartz cell, over a wavelength range of 250–500 nm. Transmission Electron Microscopy (TEM) images of the nano-capsules were obtained with a JEOL JEM–2100 microscope operating at an accelerating voltage of 200 kV. Dynamic light scattering (DLS) and Zeta potential analysis was performed using a Malvern Zetasizer Nano-ZS90 (Malvern, UK) at 25 °C with a conductivity of 0.0133 mS/cm.

### Encapsulation efficiency and release study

The loading capacity and efficiency of essential oils in the two prepared capsules were determined following the method described by Himed et al.^43^. Briefly, 10 mg of each capsule sample was dispersed in 10 mL of ethanol and shaken for 24 h to ensure complete release of the EO into the ethanol. The resulting solution was then centrifuged to separate the solid materials. The supernatant was analyzed by measuring the absorbance at wavelengths of 273 nm for EO_1_-PDA and 253 nm for EO_2_-PDA. The released amounts (µg/L) of the two essential oils were determined using Eqs. (1) and (2) for EO_1_ and EO_2_, respectively, which were derived from the calibration curves shown in Figure S3 in the supplementary information:

1 $${\rm Absorbance = 0.08712 \:conc. \:of \:EO_1-0.00721}$$

2 $${\rm Absorbance = 0.00028\: conc.\: of\: EO2\:+ 0.06573}$$

The loading capacity (or efficiency) of the essential oils was calculated using Eq. (3):

3 $$\:\mathrm{L}\mathrm{o}\mathrm{a}\mathrm{d}\mathrm{i}\mathrm{n}\mathrm{g}\:\mathrm{c}\mathrm{a}\mathrm{p}\mathrm{a}\mathrm{c}\mathrm{i}\mathrm{t}\mathrm{y}\:=\frac{M}{{M}_{o}}\mathrm{X}100$$

where *M* and *M*_*o*_ represent the weight of the loaded essential oil and the initial weight of the capsule, respectively. The release efficiency was evaluated using the same method employed for determining loading and encapsulation efficiency under buffer solution pH 5. At regular intervals, aliquots were withdrawn from the bottle for absorbance measurement to monitor EOs release from the two capsules over time.

### **Cytotoxicity evaluation using viability assay**

For the antiproliferative assays, we employed MCF-7 (human breast cancer) and HepG-2 (human hepatocellular carcinoma) cell lines, both obtained from the American Type Culture Collection (ATCC, Rockville, MD). The experimental protocol followed established methodologies by Al Zahrani et al. ^44^ and Mosmann^45^to ensure reliability. Cells (5 × 10⁴ per well) were seeded into Corning^®^ 96-well tissue culture plates and treated with eight different concentrations (3.9, 7.8, 15.6, 31.25, 62.50, 125, 250, and 500 µg/mL) of PDA, essential oils (EO1 and EO2) and their PDA nanocapsules (EO1-PDA and EO2-PDA), each in triplicate. A 0.5% DMSO control was included for comparison. After 24 h of incubation, cell viability was assessed using the MTT assay, with optical density measured at 590 nm via a microplate reader (SunRise, TECAN, Inc., USA). The IC50 values were determined by analyzing dose-response curves using GraphPad Prism software (San Diego, CA, USA). This rigorous approach underscores the potential of EOs-PDA nano-capsules in antiproliferative applications.

### Evaluation of antioxidant activity

The antioxidant activity of the free essential oils (EO1 and EO2), PDA and their polydopamine-encapsulated nanocapsules (EO1-PDA and EO2-PDA) was evaluated using the DPPH free radical scavenging method. A freshly prepared 0.004% (w/v) methanol solution of 2,2-diphenyl-1-picrylhydrazyl (DPPH) radical was stored at 10 °C in the dark. A methanol solution of each test compound was prepared with different concentration of (0.5,1, 2, 3.9, 7.8, 15.6, 31.25, 62.5, 125, 250, 500, and 1000 µg/mL), and a 40 µL aliquot was added to 3 mL of DPPH solution. Absorbance was immediately recorded at 517 nm using a UV-visible spectrophotometer (Milton Roy, Spectronic 1201) at 1-minute intervals until stabilization (16 min). The antioxidant activity of the extracts was assessed based on their redox properties, which enable them to donate hydrogen atoms or inhibit peroxide formation, leading to free radical scavenging^46^. The reduction in absorbance at 517 nm resulted in a color change from violet to colorless due to DPPH radical stabilization^47,48^. Control absorbance (without antioxidant) and ascorbic acid (reference compound) were also measured. All experiments were conducted in triplicate. The percentage inhibition (PI) of the DPPH radical was calculated using the formula:

$$\:PI=\left(\frac{AC-AT}{AC}\right)\times\:100$$

where AC is the absorbance of the control at t = 0 min, and AT is the absorbance of the sample + DPPH at t = 16 min. IC50 values were determined from dose-response curves using GraphPad Prism software (San Diego, CA, USA)^44^. The classification of IC50 values is as follows: very strong (< 10 µg/mL), strong (10–50 µg/mL), moderate (50–100 µg/mL), weak (100–250 µg/mL), and inactive (> 250 µg/mL)^49^.

### Antifungal assay

#### Minimal inhibitory concentration (MIC)

To assess the antifungal properties of EOs and PDA polymer, we employed the microdilution method to determine the Minimum Inhibitory Concentration (MIC) of the tested samples^50^. The MIC, defined as the lowest concentration that visibly inhibits fungal growth compared to control conditions, serves as a crucial measure of antifungal efficacy. This study targeted two major pathogenic fungi *Candida albicans* (PP664305) and *Pichia kudriavzevii* (PP664417) (syn. *Candida krusei*) along with an additional strain of *P. kudriavzevii* (PP664439) isolated from leukemia patients suffering from oral thrush^51^. All fungal strains were cultured and tested according to the Clinical and Laboratory Standards Institute (CLSI) broth microdilution method for yeasts (document M27). Briefly, inocula were prepared from fresh cultures, adjusted to a final concentration of 0.5–2.5 × 10^3^ CFU/mL in RPMI-1640 medium (with L-glutamine, without sodium bicarbonate), and incubated at 35 °C under standardized conditions. A stock solution of 1.0 g/mL was prepared for each tested sample in dimethyl sulfoxide (DMSO). These stock solutions were serially diluted two-fold in RPMI broth medium to obtain ten concentrations: 100, 50, 25, 12.5, 6.25, 3.125, 1.562, 0.781, 0.391, and 0.195 µg/mL. Antifungal activity was evaluated in a 96-well microplate, where 40 µL of the test sample was combined with 10 µL of fungal suspension per well. The positive control consisted of 40 µl of RPMI medium and 10 µl of fungal suspension, while the negative control included 50 µl of Sabouraud broth medium. The microplates were incubated at 37 °C for 24 h, with all experiments conducted in triplicate^52^. Following incubation, fungal growth was assessed using an Absorbance Microplate Reader (Readwell Touch Automatic ELISA Plate Analyzer, ROBONIK, India) at 630 nm. Absorbance values from EO-treated and nanopolymer treated samples were compared against untreated controls, and data were analyzed using MS Excel.

#### Fractional inhibitory concentration index

To find the way the combined EO1-PDA and EO2-PDA micelles could interact with the tested *Candida albicans* PP664305 (MG 1), *P. kudriavzevii P*P664417 (MG2) and *P. kudriavzevii* PP664439 (MG3), the checkerboard method on a 96-well microtiter plate, following the protocol described by Hlebova et al.^53^ was used. Based on the MIC determinations, the antifungal interactions of the EO1, EO2, Nano-PDA and both EOs-PDA were assessed using the fractional inhibitory concentration index (FICI), calculated as:

$$\:FICI=\frac{{MIC}_{Mixed}}{{MIC}_{1}}+\frac{{MIC}_{Mixed}}{{MIC}_{2}}$$

where MIC_Mixed_ represents the MIC of the combined EOs, MIC_1_ represents the MIC of the individual EO, and MIC_2_ represents the MIC of PDA. The results were interpreted as follows: a FICI ≤ 0.5 indicates a synergistic effect, values between 0.5 and 1.0 suggest an additive or partially synergistic effect, values between 1.0 and 4.0 indicate an indifferent effect, and a FICI > 4.0 reflects an antagonistic effect^54,55^. This approach provides a quantitative measure of interaction, determining whether the combination enhances antifungal efficacy or exhibits antagonism.

#### Transmission electron microscopy (TEM)

TEM analyses were conducted on overnight cultures of *Candida albicans* (PP664305), *Pichia kudriavzevii* (syn. *Candida krusei*, PP664417), and another strain of *P. kudriavzevii* (PP664439). The fungal cultures were treated with sublethal concentrations of 25, 3.125, and 3.125 µg/mL for the EO1-PDA nano-capsule and 3.125, 6.25, and 50 µg/mL for the EO2-PDA nano-capsule in DMSO for the three different species. Fixed cell samples were centrifuged at 2500 rpm for 10 min, washed with 0.1 mol/L MgSO_4_ buffer, and double-fixed overnight at 4 °C in a solution containing 2.5% glutaraldehyde and 1% paraformaldehyde in 0.1 mol/L cacodylate buffer supplemented with 2 mmol/L CaCl_2_ and 2 mmol/L MgCl_2_ (pH 7.2). Following fixation, the samples were washed three times with 0.1 mol/L cacodylate buffer at room temperature. The pellets were resuspended in 200 µL of 2% low-melting-point agarose and stored at 4 °C for 1 h before being cut into 1–2 mm cubes. These agar-embedded samples were treated with 2% potassium permanganate solution and incubated under gentle shaking at 4 °C for 3 h. After thorough washing with distilled water, the samples were dehydrated using a graded ethanol series (30%–100%) and embedded in Epon–Araldite resin for sectioning. Ultrathin Sect. (30 samples per treatment) were obtained using a Leica Ultracut UCT Ultramicrotome (Milan, Italy) with a diamond knife. The sections were double-stained with uranyl acetate and lead citrate before visualization under a Philips CM 10 transmission electron microscope (Japan). Image analysis was performed using ImageJ software (NIH, Bethesda, MD), enabling detailed structural evaluation of the treated fungal cells.

## Results and discussion

### GC–MS analysis of essential oils

**Fig. 2:**
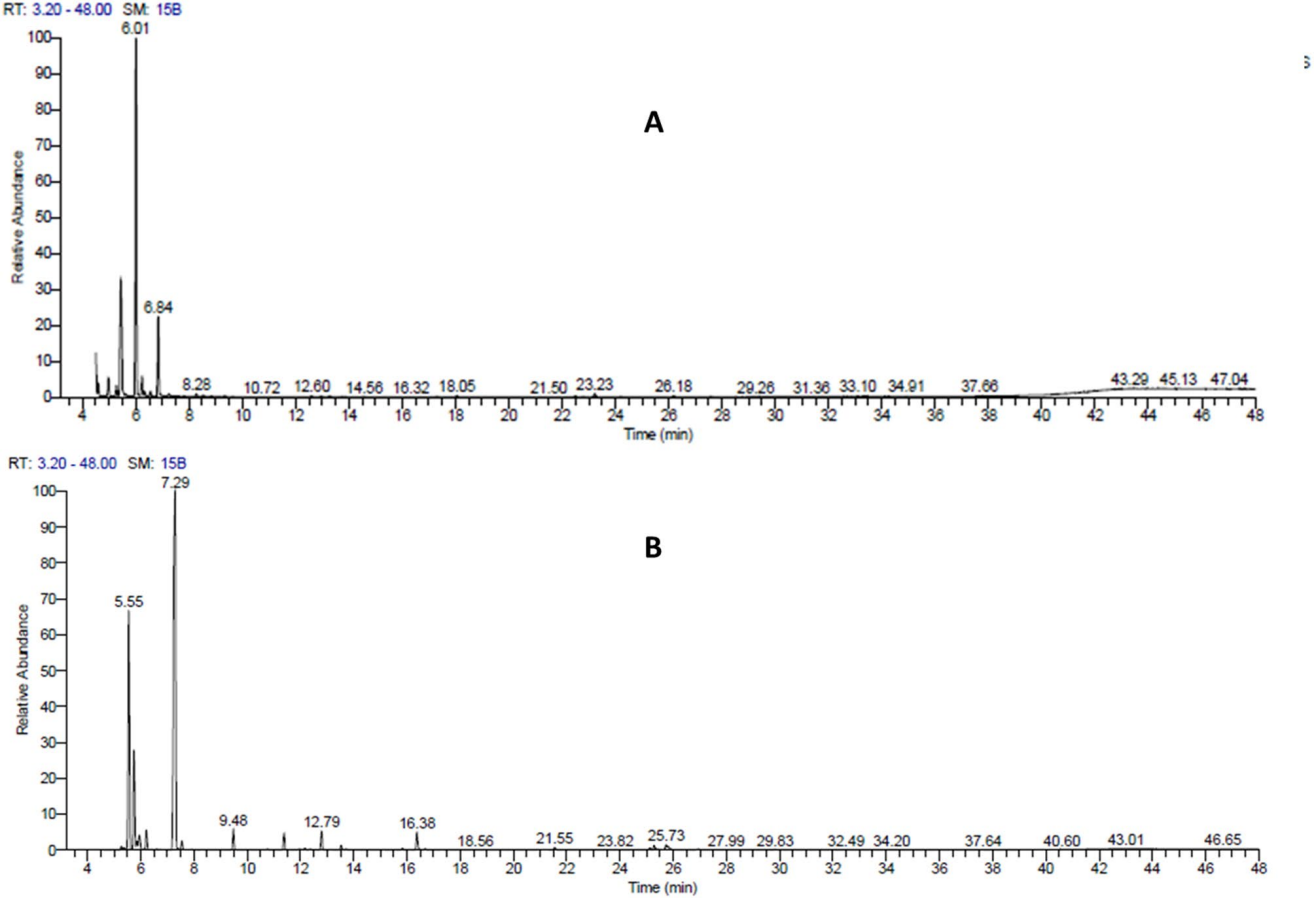
Gas chromatograph of the leaves essential oil of (**A**) *Cupressus macrocarpa* and (**B**) *Mentha longifolia*.

The extraction process yielded 5 mL of essential oil from 1.6 kg of *Cupressus macrocarpa* (0.3125% v/w) and 5 mL from 1.2 kg of *Mentha longifolia* (0.4166% v/w). The chemical composition of essential oils from *Cupressus macrocarpa* and *Mentha longifolia* was analysed using GC-MS, revealing significant differences in their volatile profiles. The results are presented in Fig. 2; Tables 1 and 2. These differences highlight the distinct characteristics and potential applications of each essential oil in medicinal, cosmetic, and aromatic industries^56^. Starting with *Cupressus macrocarpa* EO, the GC-MS analysis (Fig. 2A) identified (−)-terpinen-4-ol (52.57%) and (−)-camphor (24.98%) as the major components. Likely, Salem et al., ^31^ stated that the major chemical constituents of *Cup. macrocarpa* EO are terpinen-4-ol (23.7%), α- and D-camphor (5.4%) but in less concentration. The high concentration of (−)-terpinen-4-ol suggests strong antimicrobial and anti-inflammatory properties, as this compound is commonly associated with such bioactivities^57^. Additionally, the notable presence of (−)-camphor, α-terpineol (2.90%), and R-(+)-citronellol (11.90%) may further enhance the bioactivity and multifunctional potential of the essential oil^58^. In *Mentha* longifolia essential oil (Fig. 2B), p-Menth-4(8)-en-3-one was the dominant compound, comprising 53.77% of the oil’s total composition. This compound is known for its strong minty aroma and potential antimicrobial properties^59^. Other significant components include p-Menthan-3-one (23.30%) and cis-p-Menthan-3-one (9.16%). The presence of these compounds suggests that *Mentha longifolia* essential oil may have potent antibacterial activity and serve as a flavoring agent in food and beverages^60^. Interestingly, trace amounts of compounds such as caryophyllene (1.56%) and γ-cadinene (0.41%) indicate a complex chemical profile that may contribute to additional therapeutic effects, including anti-inflammatory properties.

**Table 1:** Chemical components (%) in the essential oil distilled from *Cupressus macrocarpa*.

No.	RT	Compound	% of compound
1	4.51	β-linalool	0.30
2	4.59	β -terpineol	1.64
3	4.98	4-Isopropyl-1-methyl-2-cyclohexen-1-ol	2.83
4	5.28	4-Isopropyl-1-methyl-2-cyclohexen-1-ol	1.56
5	5.46	(−)-camphor	24.98
6	6.01	(−)-Terpinen-4-ol	52.57
7	6.23	α-terpineol	2.90
8	6.34	trans-(−)-p-Menth-1-en-3-ol	0.58
9	6.56	cis-(−)-p-menth-1-en-3-ol	0.74
10	6.84	R-(+)-Citronellol	11.90

**Table 2:** Chemical components (%) in the essential oil distilled from *Mentha longifolia*.

No.	RT	Compound	% of compound
1	5.27	4(10)-Thujen-3-ol	0.32
2	5.56	p-Menthan-3-one	23.30
3	5.75	cis-p-Menthan-3-one	9.16
4	5.86	L-(-)-Menthol	0.75
5	5.95	trans-p-Menth-8-en-3-one	1.68
6	6.21	p-menth-1-en-8-ol	1.85
7	7.29	p-Menth-4(8)-en-3-one	53.77
8	7.55	p-Menth-1-en-3-one	0.70
9	9.48	2-Pinen-4-one	1.98
10	11.39	Caryophyllene	1.56
11	12.79	β-Cubebene	1.80
12	13.53	γ-Cadinene	0.41
13	16.38	.tau.-Cadinol	1.80
14	25.28	Oleic acid, methyl ester	0.47
15	25.73	Labd-14-ene-8,13-diol, (13R)-	0.44

### Synthesis and characterization of PDA and EO-PDA capsules

#### FTIR analysis

**Fig. 3:**
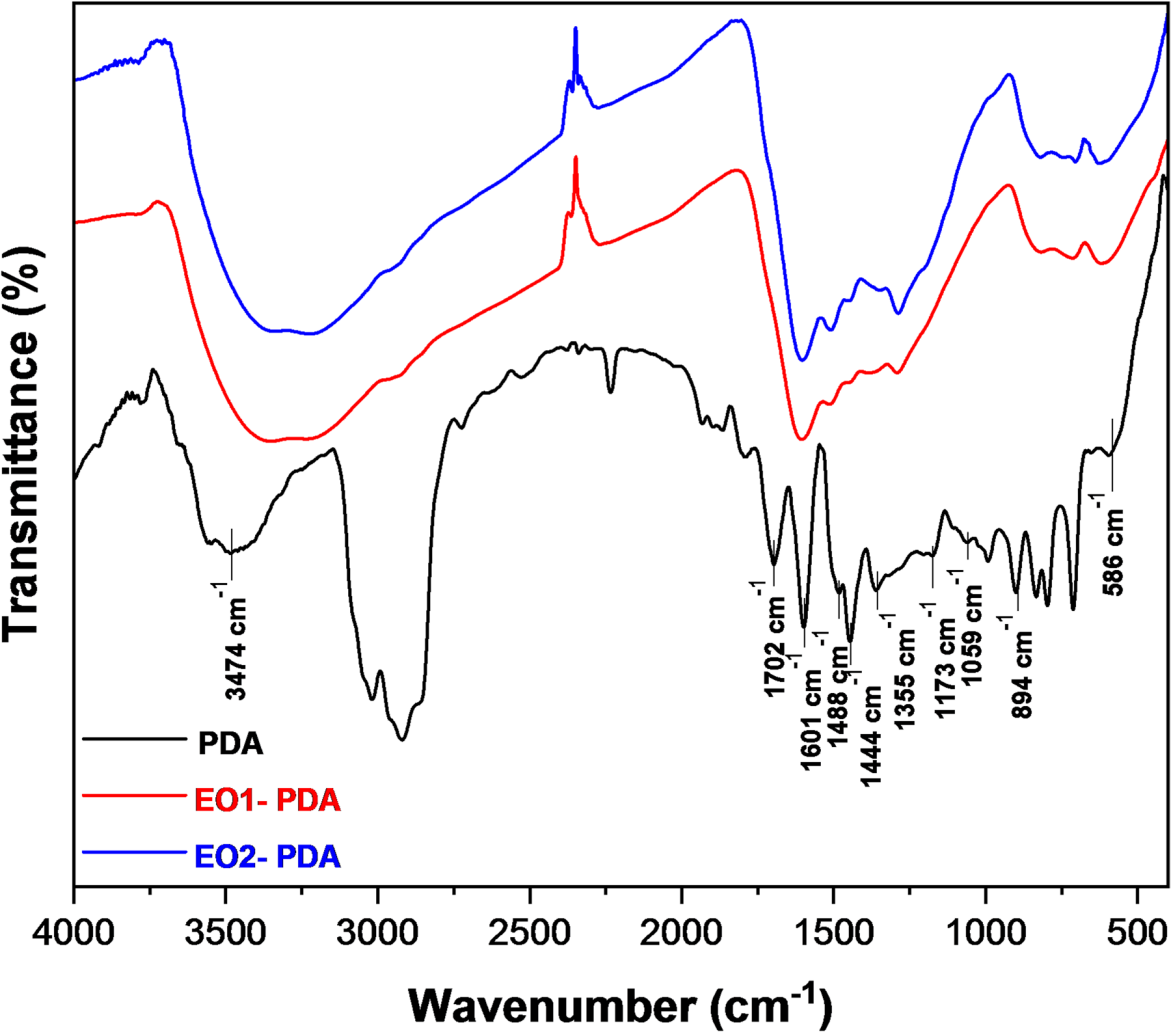
FTIR spectrum of PDA, EO1-PDA, and EO2-PDA.

Polydopamine was successfully synthetized by oxidative self-polymerization of dopamine HCl monomer in an alkaline condition as a dark brown powder with a yield of 0.45 g. The FTIR spectrum of polydopamine was presented in Fig. 3. The results indicate a broad absorption peak at 3474 cm^− 1^ corresponds to O-H and N-H stretching vibrations, indicative of hydroxyl and amine groups, with strong hydrogen bonding. The sharp peak at 1702 cm^− 1^ is attributed to C = O stretching, suggesting the presence of carbonyl groups likely formed during dopamine polymerization. Peaks at 1601 cm^− 1^ and 1488 cm^− 1^ are associated with C = C aromatic ring stretching and N-H bending vibrations, respectively, highlighting the aromatic and amine functionalities. Additional peaks at 1444 cm^− 1^ and 1355 cm^− 1^ correspond to C-H bending and C-N stretching, confirming the presence of aromatic and amine groups. The peak at 1173 cm^− 1^ is assigned to C-O stretching, characteristic of catechol groups, while the band at 1059 cm^− 1^ relates to C-O-C or C-OH bending vibrations. Finally, peaks in the range of 894–586 cm^− 1^ represent out-of-plane aromatic C-H bending vibrations, reinforcing the aromatic nature of the polymer^61,62^. These findings confirm the successful polymerization of dopamine into polydopamine.

The FTIR spectra of the isolated essential oils, EO1 and EO2, are illustrated in Figure S1 and Figure S2 in the Supplementary Information. Both spectra exhibit characteristic absorption bands related to their functional groups. Prominent C–H stretching vibrations appear in the range of 2920–2960 cm^− 1^, while sharp C = O stretching bands near 1700 cm^− 1^ indicate the presence of aldehydes, ketones, or esters. In the 1000–1600 cm^− 1^ region, strong C = C and C–O stretching bands are observed, typical of aromatic and oxygenated compounds commonly found in plant-derived essential oils. These spectral features confirm the complex organic composition of EO1 and EO2. Following encapsulation, the spectra of EO1-PDA and EO2-PDA nanocapsules displayed in Fig. 3 reveal the presence of both PDA and essential oil functional groups, along with noticeable shifts in peak positions and intensity. The broad O–H/N–H stretching band of PDA near 3474 cm^− 1^ shifts slightly and decreases in intensity, suggesting hydrogen bonding interactions between PDA and essential oil constituents. The C = O stretching peak of the essential oils also shifts and broadens in the nanocapsules, further indicating interaction and entrapment within the PDA matrix. These spectral changes confirm successful encapsulation of the essential oils, likely involving hydrogen bonding and π–π interactions between the oils and PDA, rather than simple surface adsorption.

#### TEM analysis of EO‏-‏PDA

**Fig. 4:**
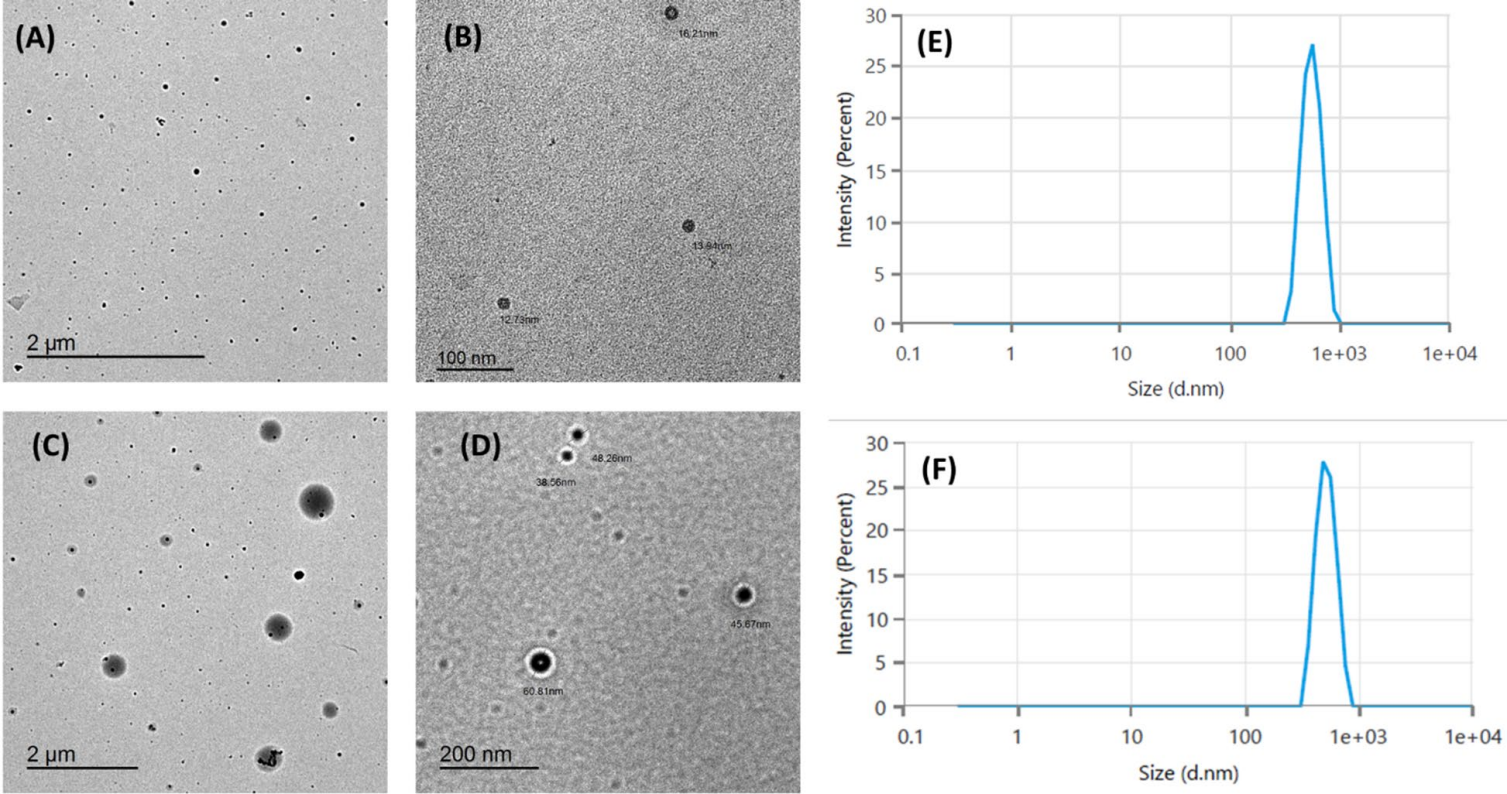
TEM images of EO1-PDA (**A** and **B**) and EO2-PDA (**C** and **D**). Dynamic Light Scattering (DLS) of EO1-PDA(**E**) and EO2-PDA (**F**).

The TEM images of EO1‏-‏PDA (Figs. 4A and B) and EO2‏-‏PDA (Figs. 4C and D) provide significant insights into the morphology, size, and structural characteristics of the encapsulated systems^63–65^. The observed dark and uniform outer layers suggest successful deposition of the polydopamine coating around the essential oil cores. Polydopamine’s ability to self-polymerize under mild oxidative conditions likely contributed to the uniform encapsulation observed. This coating not only stabilizes the essential oil droplets but also protects them from environmental degradation, such as oxidation or volatilization, which are common issues with essential oils^66^. Additionally, the EO1-PDA and EO2-PDA systems exhibited average particle sizes of 28.70 ± 9.11 nm and 47.36 ± 14.61 nm, respectively, as determined from TEM images (Fig. 4A and C), confirming their formation as spherical nanoparticles. These measurements were based on randomly analyzing more than 100 individual particles using ImageJ software. Both small and relatively larger particles visible in Fig. 4C and D were included to minimize bias in the average size determination. The corresponding particle size distribution is illustrated in Figure S4 in supplementary information. The presence of a few larger aggregates is attributed to the coalescence of oil droplets during drying and is consistent with the polydisperse nature later observed by DLS analysis.

#### Dynamic light scattering and zeta potential

The encapsulation of essential oils with polydopamine was evaluated through dynamic light scattering (DLS) and zeta potential measurements, providing insights into particle size and surface charge, key indicators of stability and potential functionality in colloidal systems. The DLS analysis revealed that the Z-average hydrodynamic diameters of the nanocapsule systems were 582.30 ± 39.60 nm for EO1-PDA and 547.60 ± 19.33 nm for EO2-PDA (Fig. 4E and F). Additionally, the polydispersity index (PDI) was found to be 0.8781 ± 0.33 for EO1-PDA and 0.6846 ± 0.27 for EO2-PDA, indicating a moderately broad particle size distribution. These results suggest some variability in the size of the nanocapsules, which may be attributed to the nature of the essential oil components and their interactions during encapsulation. The size distribution suggests that the encapsulation process was consistent and produced particles suitable for applications requiring nanometer-scale formulations, such as drug delivery or controlled release systems^67^.

Although DLS measurements typically yield larger sizes than TEM, the nearly 10-fold difference observed here can be attributed to several factors. First, DLS measures the hydrodynamic diameter, which includes the hydrated PDA shell and any solvent layers or weakly associated aggregates present in the dispersion. Second, the relatively high PDI values suggest a wide range of particle sizes and partial aggregation in aqueous solution, which contribute disproportionately to light-scattering intensity and increase the apparent size. In contrast, TEM measures the dry core size under vacuum, where dehydration and shrinkage occur. These complementary results highlight the importance of interpreting both techniques together when evaluating nanoparticle behavior in hydrated and dry states^68,69^.

In addition, the zeta potential values were measured as −33.15 mV for EO1-PDA and − 31.29 mV for EO2-PDA. These negative values indicate that the particles possess sufficient electrostatic repulsion to maintain colloidal stability, thereby minimizing the likelihood of aggregation. Both values exceed the commonly accepted threshold of ± 30 mV, which is often associated with electrostatically stable nanoparticle suspensions. This suggests that the nanocapsules are likely to remain well-dispersed under the conditions studied^70^. It is important to note that DLS and zeta potential analyses were performed only for the EO-loaded PDA nanocapsules (EO1-PDA and EO2-PDA). Measurements for unloaded PDA or pure essential oils were not conducted, as the primary objective of this study was to evaluate the physicochemical characteristics and stability of the encapsulated nanostructures.

#### Encapsulation efficiency and release behavior of EO–PDA nanocapsules

The encapsulation efficiency of EO1 and EO2 in polydopamine nanocapsules was determined to evaluate the effectiveness of the loading process. The calculated encapsulation efficiencies were 23% for EO1 (*Cupressus macrocarpa* oil) and 73.69% for EO2 (*Mentha longifolia* oil). These results suggest that EO2 was more effectively encapsulated within the polydopamine matrix compared to EO1, likely due to differences in chemical composition, polarity, or affinity for the PDA surface. The relatively high loading efficiency of EO2 indicates a favorable interaction with the PDA structure, potentially enhancing its stability and sustained bioactivity. Overall, these findings confirm that the essential oils were successfully encapsulated within the polydopamine nanocapsules rather than merely adsorbed on the surface.

**Fig. 5:**
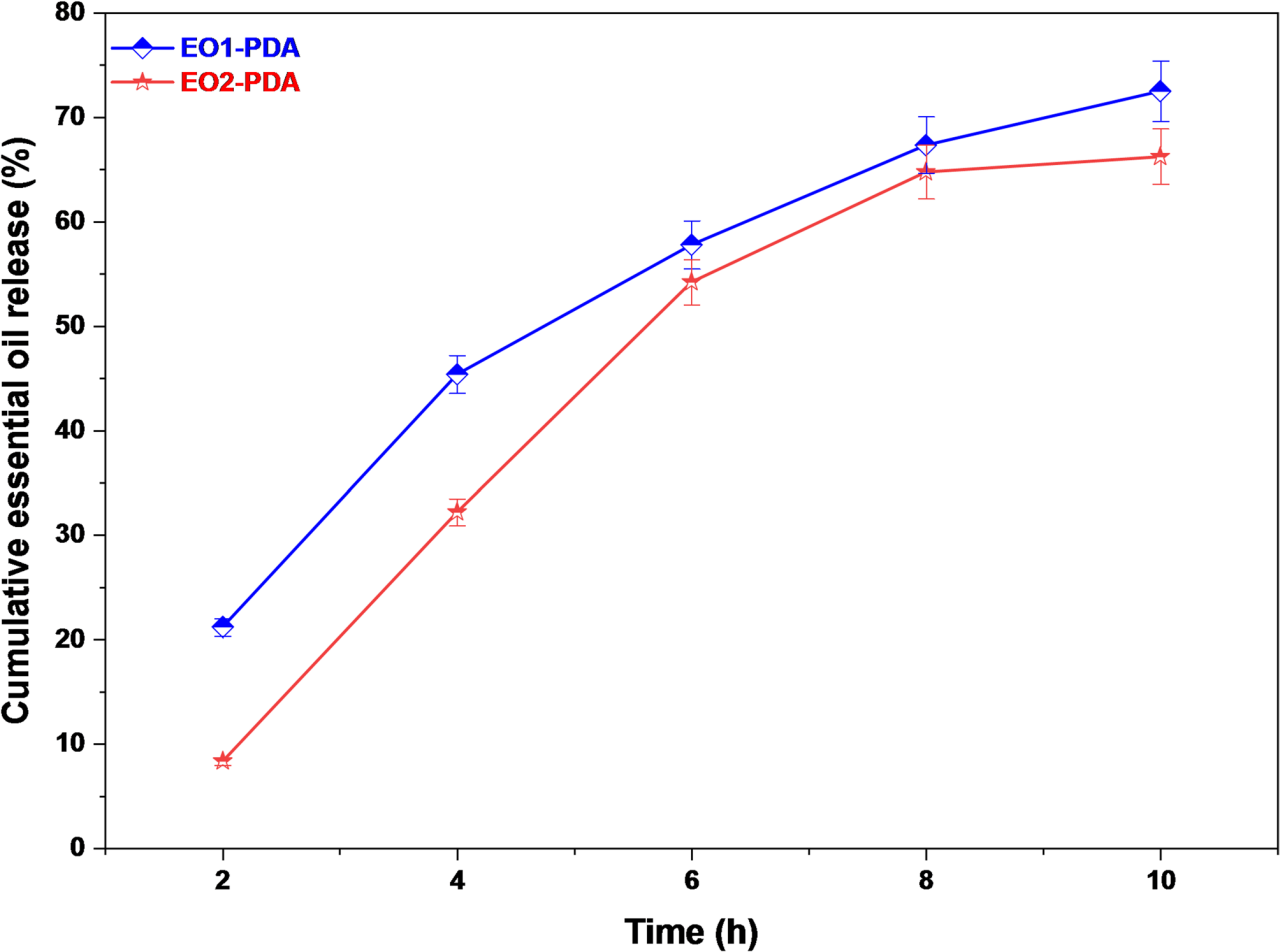
In vitro cumulative release profiles of essential oils encapsulated in polydopamine nanocapsules (EO1-PDA and EO2-PDA) as a function of time.

In addition to loading and encapsulation efficiency, the release behavior of the EOs-PDA nanocapsules was investigated to evaluate their ability to provide controlled delivery. Figure 5 presents the in vitro cumulative release profiles of EO1-PDA and EO2-PDA over 10 h. Both systems exhibit a sustained and time-dependent release pattern, indicating effective confinement of the EOs within PDA matrix. An initial release phase is observed during the early stage of the experiment, which can be attributed to the diffusion of essential oil molecules weakly bound to PDA nanocapsule. This initial release is followed by a slower and more controlled release stage, suggesting that the majority of the essential oil is encapsulated within the PDA shell and released gradually through diffusion and matrix relaxation. Notably, EO1-PDA shows a slightly higher cumulative release compared to EO2-PDA throughout the release period. This difference may be related to variations in the molecular structure, volatility, and affinity of the essential oils toward the PDA network. Overall, these findings confirm that PDA nanocapsules not only achieve efficient loading and encapsulation of essential oils but also provide a sustained release profile, enhancing their potential applicability in controlled delivery systems.

### Cytotoxicity evaluation

**Fig. 6:**
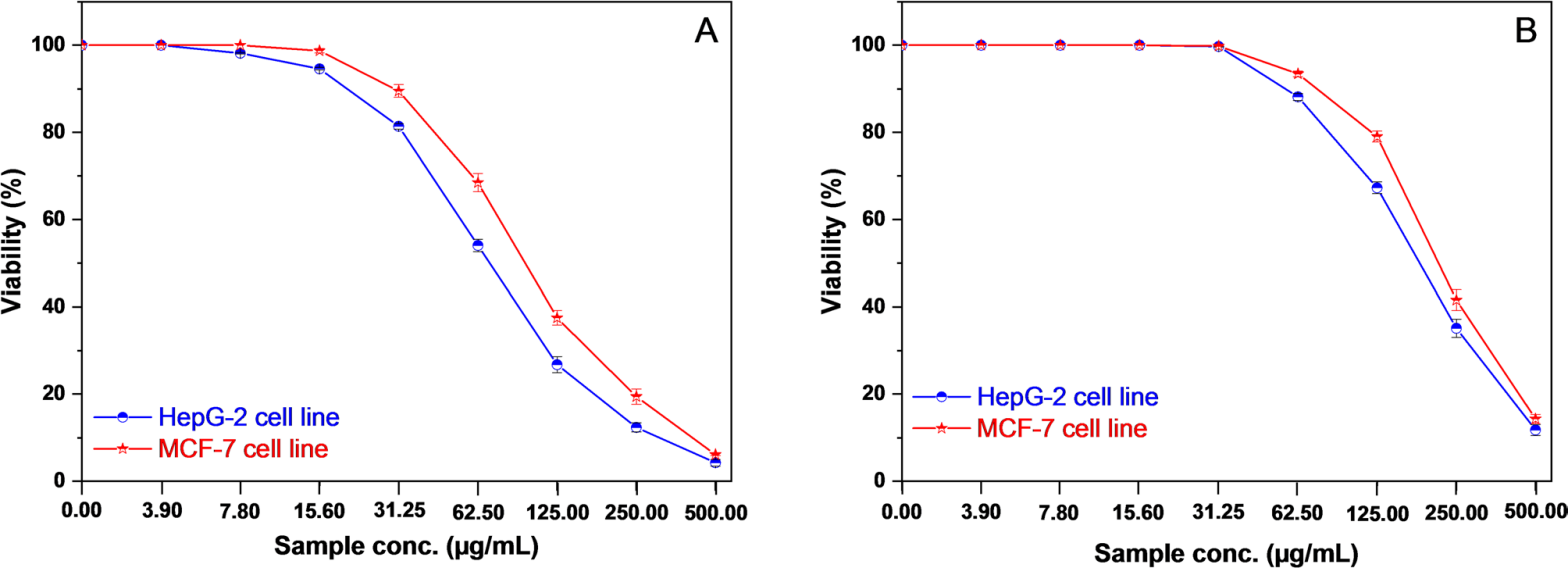
Evaluation of cytotoxicity against HepG-2 cell line and MCF-7 cell line for (**A**) EO1- PDA nano-capsule and (**B**) EO2- PDA nano-capsule.

Cytotoxicity was expressed as the concentration that caused a 50% reduction in cell viability (IC_50_). This assay was conducted to evaluate the effect of free *Cupressus macrocarpa* oil (EO1), *Mentha longifolia* oil (EO2), blank PDA and their corresponding PDA-encapsulated formulations with PDA on MCF-7 cells (human breast cancer cell line) and HepG-2 cells (human hepatocellular carcinoma cell line). The data, obtained in triplicate for each concentration, are plotted (Figure S5 – S6 in supplementary information and Fig. 6), and the IC_50_ values (the concentrations required to inhibit 50% of cell viability) were determined. The free EO1 exhibited strong cytotoxicity, with IC₅₀ values of 6.45 ± 0.32 µg/mL against HepG-2 and 8.25 ± 0.31 µg/mL against MCF-7 (Figure S5.A). In comparison, free EO2 showed moderate cytotoxicity, with IC₅₀ values of 30.97 ± 0.89 µg/mL and 51.54 ± 1.34 µg/mL for the same respective cell lines (Figure S5.B). These findings indicate that EO1 was more potent than EO2 and that HepG-2 cells were generally more sensitive than MCF-7 cells to both oils. The cytotoxicity of blank PDA nanocapsules was also evaluated to assess the contribution of the carrier material. PDA exhibited relatively low cytotoxicity toward both cell lines, with IC₅₀ values of 219.64 ± 7.05 µg/mL for HepG-2 cells and 245.26 ± 6.28 µg/mL for MCF-7 cells (Figures S6 in supplementary information). These results confirm that PDA alone shows limited cytotoxic effects at concentrations significantly higher than those required for the free essential oils.

Upon encapsulation, the cytotoxic effects remained evident but required higher concentrations. The EO1-PDA nano-capsule exhibited strong cytotoxicity against both HepG-2 and MCF-7 cells, with an IC_50_ of 71.80 ± 1.98 µg/mL for HepG-2 cells and 99.79 ± 3.14 µg/mL for MCF-7 cells (Fig. 6.A). The viability curves indicate a steep decline in cell survival with increasing concentration, suggesting potent inhibitory activity. Similarly, as illustrated in Fig. 6.B, the EO2-PDA nano-capsule demonstrated cytotoxic effects, albeit at higher concentrations, with an IC_50_ of 192.22 ± 5.21 µg/mL for HepG-2 cells and 221.90 ± 6.03 µg/mL for MCF-7 cells. The viability curves for EO2-PDA show a more gradual decrease, indicating a lower cytotoxic potency compared to EO1-PDA. Importantly, the cytotoxicity of the EO–PDA formulations was markedly higher than that of PDA alone, indicating that the observed biological activity is primarily driven by the encapsulated essential oils rather than the carrier material.

Although the IC₅₀ values for the encapsulated forms were higher compared to their free oil counterparts likely due to a sustained release mechanism, the formulations still demonstrated notable cytotoxic activity. Overall, these results confirm that EO1-PDA was the most cytotoxic formulation, particularly toward HepG-2 cells, suggesting greater therapeutic potential. The observed differences between the two cell lines may reflect variations in cellular uptake, membrane permeability, or intrinsic sensitivity to the bioactive compounds. These findings support the potential application of EO-PDA nanocapsules as controlled-release systems for anticancer therapies.

### Antioxidant activity against DPPH

**Fig. 7:**
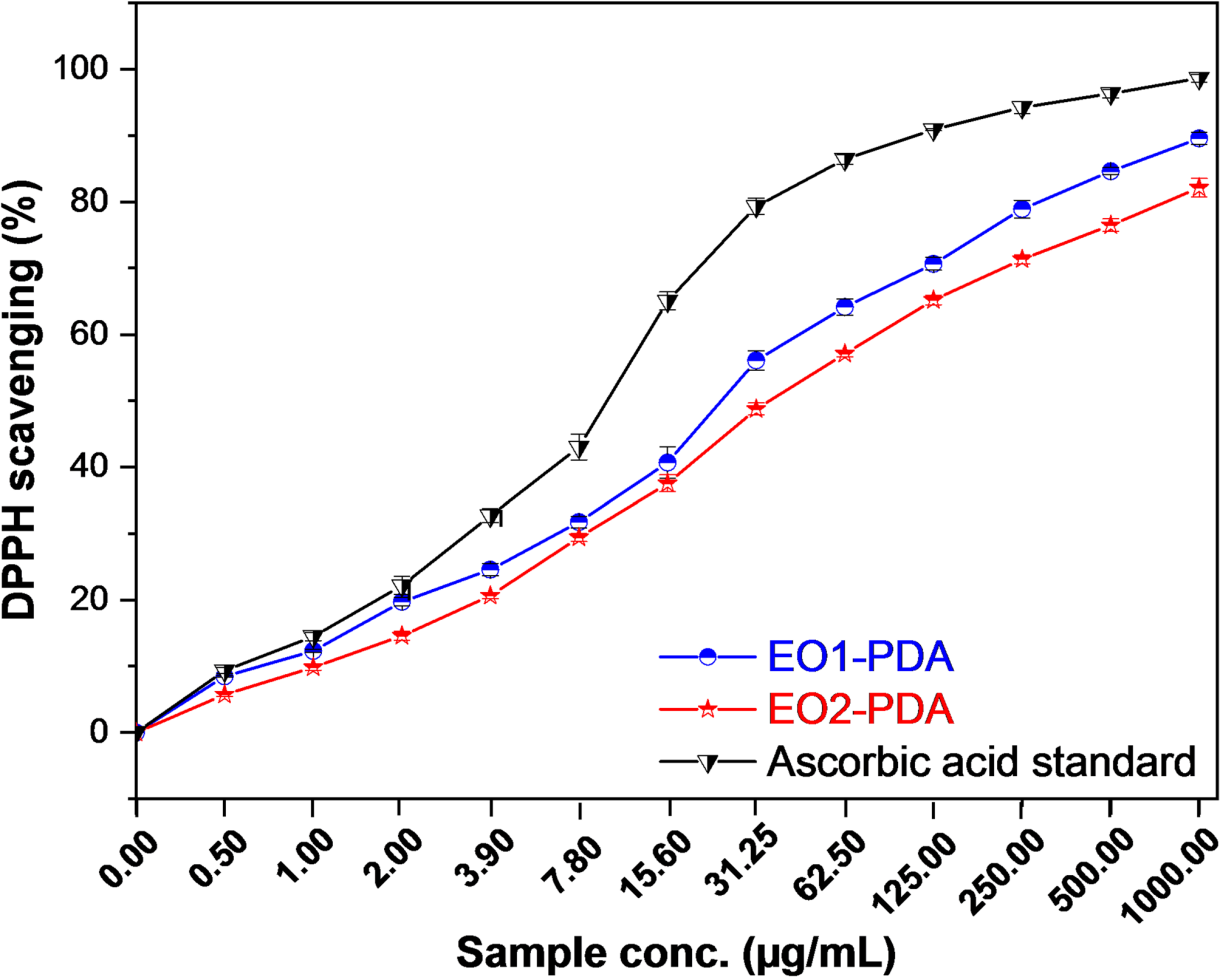
Evaluation of Antioxidant Activity using DPPH scavenging of EO1- PDA nano-capsule, EO2- PDA nano-capsule and ascorbic acid as standard.

The antioxidant properties of EO1-PDA and EO2-PDA nano-capsules were evaluated using the DPPH scavenging assay to assess their ability to neutralize free radicals. In addition, blank PDA nanocapsules were tested to assess the intrinsic antioxidant contribution of the carrier material. Their performance was compared to ascorbic acid, a well-known antioxidant, across a range of concentrations. As illustrated in Fig. 7 and Figure S7 in supplementary information, all tested samples exhibited a concentration-dependent increase in DPPH scavenging activity. Ascorbic acid demonstrated the highest antioxidant efficiency, reaching nearly complete scavenging at higher concentrations, with an IC_50_ of 10.21 ± 0.77 µg/mL. Blank PDA nanocapsules showed weak antioxidant activity, with an IC_50_ of 101.69 ± 3.73 µg/mL. In comparison, both EO1-PDA and EO2-PDA displayed strong antioxidant potential with an IC_50_ of 25.04 ± 1.08 µg/mL and 35.94 ± 1.29 µg/mL, respectively. These results highlight the significant antioxidant properties of EOs-PDA nano-capsules, indicating that they maintain stability due to successful drug entrapment. When compared to their free forms (Figure S7 in supplementary information), EO1 (IC₅₀ = 29.35 ± 1.06 µg/mL) and EO2 (IC₅₀ = 13.91 ± 0.75 µg/mL) showed that encapsulation slightly enhanced the activity of EO1 but reduced that of EO2. This difference may be attributed to interactions between the essential oils and the PDA matrix, which may affect the release and availability of antioxidant constituents.

The markedly lower IC₅₀ values of EO1-PDA and EO2-PDA compared to blank PDA indicate that the antioxidant effect predominantly originates from the essential oils. In addition, the strong antioxidant activity of the EOs-PDA nano-capsules, with IC_50_ values less than 50 µg/mL underscores their effectiveness. This efficacy is largely attributed to the high levels of monoterpenoid constituents found in the formulation and the contribution of PDA, which enhances the radical scavenging ability^71–73^. The scavenging curves indicate that while both EO1-PDA and EO2-PDA nano-capsules effectively reduced free radicals, their activity was lower than that of ascorbic acid. Among the essential oil-loaded nano-capsules, EO1-PDA exhibited superior antioxidant activity, suggesting a greater ability to donate electrons or neutralize reactive species. This difference may be attributed to variations in the chemical composition of the encapsulated essential oils, particularly the concentration and type of bioactive monoterpenoids present. Given these compelling findings, EOs-PDA nano-capsules hold significant promise for enhancing the treatment of oxidative stress-related conditions, particularly in applications where natural antioxidant formulations are preferred. Their dose-dependent radical scavenging suggests they could be effective in therapeutic strategies for oxidative stress-related conditions, including oral thrush infections, where oxidative damage contributes to disease progression.

### Antifungal activity of essential oils

#### Essential oils and PDA antifungal activity

To comprehensively evaluate the antifungal activity of essential oils (EOs) and polydopamine (PDA) against *Candida albicans* (PP664305) and *Pichia kudriavzevii* (PP664417 and PP664439), a microdilution method was employed, using saline as a negative control and an inoculum/medium as a positive control. This method ensures accurate quantification of the antifungal efficacy of the tested compounds by directly comparing absorbance values between treated and untreated wells. Table 3 demonstrates the significant antifungal potential of EO1 and EO2, exhibiting a concentration-dependent inhibitory effect on fungal growth. EO1 (*Cupressus macrocarpa* oil) displayed notable antifungal activity, particularly against *P. kudriavzevii* strains, with minimum inhibitory concentration (MIC) values of 50 µg/L for *C. albicans* PP664305, 25 µg/L for *P. kudriavzevii* PP664417, and 12.5 µg/L for *P. kudriavzevii* PP664439. These results suggest that EO1 is more effective against *P. kudriavzevii* than *C. albicans*, reinforcing the notion that essential oils exhibit strain-specific activity. The potent antimicrobial action of EO1 is attributed to its rich composition of camphor, α-terpineol, and R-(+)-citronellol, which are known for their strong antifungal and anti-inflammatory properties. Camphor, in particular, has been reported as an effective anticandidal terpenoid, capable of reducing *C. albicans* activity. Additionally, terpinenes and terpenoids enhance membrane permeability, amplifying the antifungal effects through increased intracellular penetration^74–77^.

EO2 (*Mentha longifolia* oil) exhibited an even stronger inhibitory effect, as indicated by its lower MIC values. Specifically, EO2 demonstrated greater inhibition against *C. albicans* PP664305, with an MIC of 50 µg/L, and showed remarkable efficacy against *P. kudriavzevii* strains, with an MIC of 12.5 µg/L. This enhanced antifungal activity is likely due to the presence of p-Menthan-3-one, cis-p-Menthan-3-one, and menthol, all of which contribute to antimicrobial action. Menthol, in particular, has been suggested as a potential anticandidal agent and has been shown to enhance conventional antifungal treatments against resistant strains such as *C. glabrata* and *C. krusei* (*P. kudriavzevii*)^78^. These findings underscore the superior antifungal potential of *M. longifolia* oil and highlight its promising application as an alternative treatment for fungal infections Table 3.

Antifungal activity of essential oils EO1 (*Cupressus macrocarpa*) and EO2 (*Mentha longifolia*), nano- Polydopamine (Nano-PDA) and their combinations (EO1–PDA, EO2–PDA). The interactions were evaluated using the fractional inhibitory concentration index (FICI) against tested species.

**Table 3:** Antifungal activity of essential oils EO1, EO2, Nano-PDA, and their combinations (EO1–PDA, EO2–PDA) against *Candida albicans* PP664305 (MG1), *P. kudriavzevii* PP664417 (MG2) and *P. kudriavzevii* PP664439 (MG3). The interactions were evaluated using the fractional inhibitory concentration index.

Species	MICs					FICIc	IN	FICIm	IN
	EO1	EO2	Nano-PDA	EO1-PDA	EO2-PDA				
MG1	100.00	50.00	25.00	25.00	3.125	1.25	Inf	0.188	Syn
MG2	50.00	25.00	50.00	12.50	12.50	0.50	Syn	0.750	Psy
MG3	25.00	12.50	25.00	12.50	6.25	0.50	Syn	0.750	Psy

Syn; synergistic (FICI ≤ 0.5), Psy; partially synergistic (0.5 < FICI ≤ 0.75), Inf; indifferent (FICI ≥ 2), IN; interaction, FICIc; fractional inhibitory concentration index of EO1-PDA, FICIm; fractional inhibitory concentration index of EO1-PDA.

Furthermore, the data presented in Table 3 confirm the significant antifungal efficacy of PDA against *C. albicans* and *P. kudriavzevii*. The MIC values for PDA were 25 µg/mL for ***C.*** *albicans* PP664305, 50 µg/mL for *P. kudriavzevii* PP664417, and 25 µg/mL for *P. kudriavzevii* PP664439, demonstrating potent inhibitory effects across all tested fungal strains. These results reinforce the antimicrobial potential of PDA as a novel antifungal agent. The inhibitory mechanism of PDA may be attributed to their potential to disrupt fungal cell membranes and interfere with biofilm formation as suggested by previous studies on PDA nano capsule systems^36,79,80^. Previous studies have shown that PDA-modified nanoparticles can serve as highly effective drug carriers, facilitating controlled release of antimicrobial agents while enhancing their stability and bioavailability. This strategy has been successfully applied in biomedical applications, such as reducing microbial adhesion on catheters, thereby improving patient safety and treatment outcomes^36,79,80^. The significant activity observed in this study supports the further development of PDA-based formulations for antifungal therapies.

#### EOs ‏-PDA nano-capsules antifungal activity

Essential oils (EOs) are well known for their potent antifungal activity; however, their inherent instability and volatility can limit their effectiveness and shelf life. Consequently, nanoencapsulation using polydopamine (PDA) offers a strategy to overcome these limitations by protecting EO constituents and enhancing their controlled release and interaction with microbial cells .Thus, Nano-encapsulation using polydopamine (PDA) offers a strategy to protect EOs from degradation, improve dispersion, and potentially enhance antifungal performance.

As summarized in Table 3, both EO1 (Cupressus macrocarpa) and EO2 (Mentha longifolia) displayed notable inhibitory effects against Candida albicans (PP664305) and Pichia kudriavzevii strains (PP664417 and PP664439), with MIC values ranging between 12.5 and 100 µg/mL. Nano-polydopamine (PDA) alone exhibited moderate antifungal activity between 25 and 50 µg/mL., confirming its inherent bioactivity and potential as a functional carrier. Remarkably, when EOs were incorporated into PDA nanoparticles (EO–PDA), a pronounced reduction in MIC values was observed across all tested strains, than either the free EO or PDA, indicating enhanced antifungal potency following encapsulation.

Encapsulation of EO1 reduced the MIC from 100 µg/mL to 25 µg/mL against C. albicans and from 50 µg/mL to 12.5 µg/mL against P. kudriavzevii strains. Similarly, EO2–PDA nanoformulations exhibited even greater efficacy, with MIC values decreasing from 50 to 12.5 µg/mL for the free EO2 to 3.125–12.5 µg/mL after encapsulation. The corresponding fractional inhibitory concentration index (FICI) values confirmed synergistic (≤ 0.5) or partially synergistic interactions between EO components and PDA matrices, suggesting that PDA contributes to antifungal enhancement rather than acting merely as an inert carrier.

These results demonstrate that encapsulation of EOs within PDA nanoparticles significantly enhances antifungal efficacy, likely due to improved stability, controlled release, and increased interaction with fungal cells. The observed synergy between PDA and EO bioactives suggests that PDA acts not only as a protective carrier but also as a functional component contributing to the overall antifungal performance. Thus, PDA-based nanoencapsulation not only stabilizes essential oils but also amplifies their antifungal performance through synergistic mechanisms. To investigate how the combined microcapsules EO1-PDA and EO2-PDA interact with the tested strains of *Candida albicans* (PP664305, MG1), *P. kudriavzevii* (PP664417, MG2), and *P. kudriavzevii* (PP664439, MG3), we determined the Fractional Inhibitory Concentration Indices (FICI) for each combination. The results presented in Table 3 aiso provide a comprehensive evaluation of the antifungal interactions between EOs and PDA nanoparticles against *Candida albicans* (PP664305, MG1) and *Pichia kudriavzevii* (PP664417, MG2; PP664439, MG3). These results highlight the potential of EO-PDA nano-capsules to enhance the antifungal efficacy of the individual EOs, particularly through synergistic and partially synergistic effects. For EO1, the combination with Nano-PDA demonstrated a synergistic effect (FICI ≤ 0.5) against *P. kudriavzevii* strains (MG2 and MG3), significantly lowering the MIC required for effective fungal inhibition. However, against *C. albicans* (MG1), the EO1-PDA combination showed an indifferent interaction (FICI = 1.25), suggesting that EO1 alone or in combination with Nano-PDA may have limited effectiveness against this strain. This could be attributed to the composition of EO1, where the presence of camphor, α-terpineol, and R-(+)-citronellol contributes to its antifungal activity, yet may not be sufficient to induce a strong interaction with *C. albicans*. In contrast, EO2 exhibited superior antifungal potential, showing a synergistic effect (FICI = 0.188) against *C. albicans* (MG1). This suggests that EO2, when combined with Nano-PDA, enhances its ability to disrupt fungal growth mechanisms in *C. albicans*. Moreover, EO2-PDA displayed partially synergistic effects (FICI = 0.75) against *P. kudriavzevii* strains (MG2 and MG3), reinforcing the effectiveness of EO2 as an antifungal agent. The presence of p-Menthan-3-one and cis-p-Menthan-3-one in EO2 is likely responsible for these strong antifungal effects, particularly when enhanced by the adhesive properties of Nano-PDA. The overall findings suggest that the encapsulation of EOs with Nano-PDA enhances their activity against pathogenic fungi. The significant synergistic interaction of EO2-PDA with *C. albicans* underscores its potential as an effective antifungal agent, whereas EO1-PDA was more effective against *P. kudriavzevii*. These differences in efficacy highlight the importance of EO composition and fungal strain susceptibility in determining antifungal activity. The results further suggest that EO-PDA nano-capsules can be tailored for specific fungal infections, offering a promising strategy for improving antifungal treatments.

**Fig. 8:**
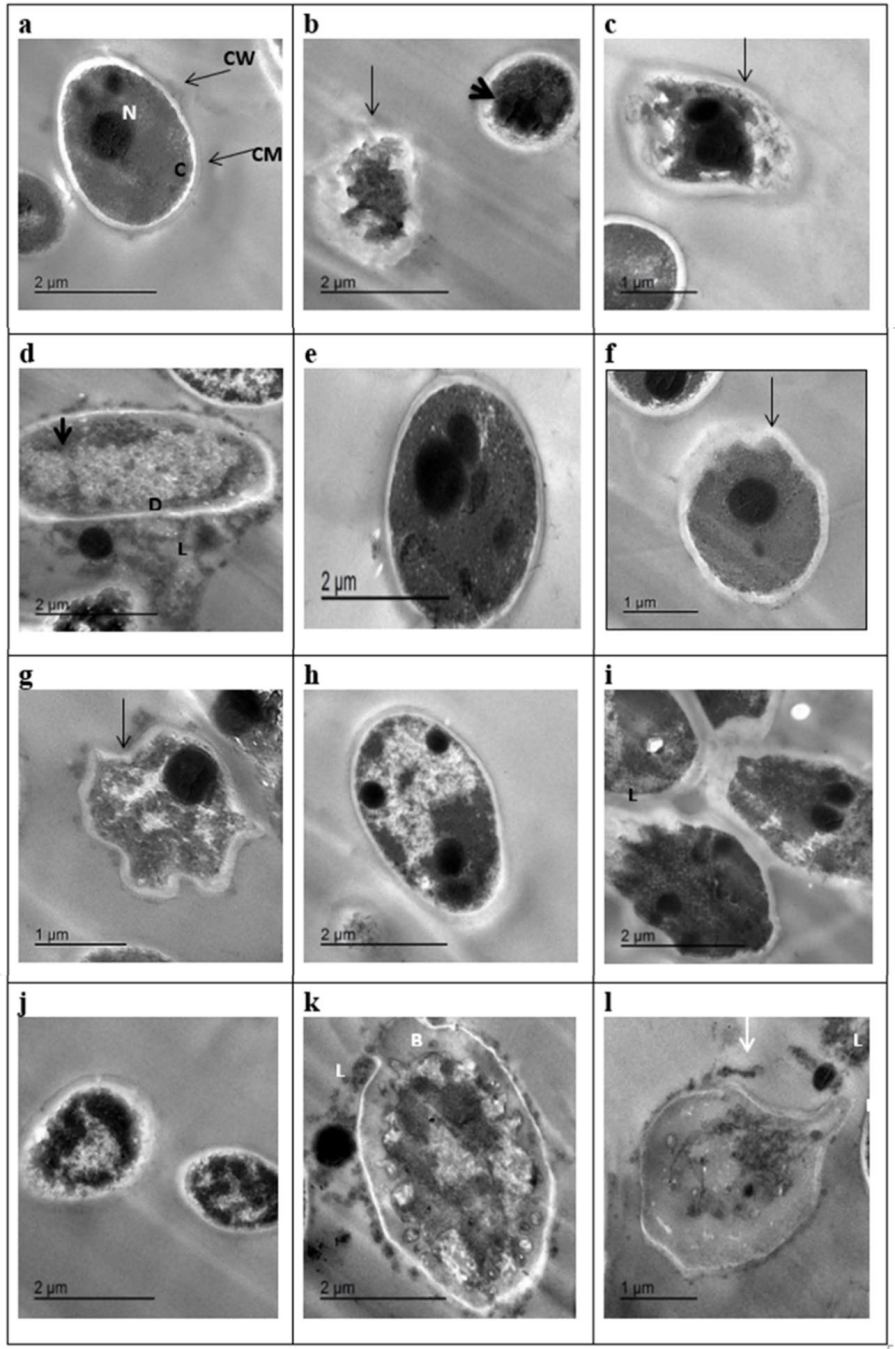
a-d, TEM micrographs of *C. albicans* PP664305 (MG 1): a; untreated cells, normal ovoid, intact cell wall (CW), regular cell membrane (CM), intact nucleus (N), cytoplasm (C). Treated cells; b: ‏EO1-PDA (apoptotic cell bursting, arrow; membrane irregularity, arrow head)، c: EO2 –PDA (necrotic cell) ‏,‏ d;‏ degraded outer wall layer ‏(D) leaked cytoplasm and nucleus (L)‏; disrupted cytoplasm ‏(arrowhead).‏ ‏ e-g, *P. kudriavzevii* PP664417 (MG 2): e, normal cell. f, g; treated cells‏: f, EO1 –PDA (early apoptotic cell with condensed chromatin arrow),‏ g,‏ ‏EO2-PDA (elder apoptotic cell with less condensed cytoplasm, irregular shape). h-l, ‏*P. kudriavzevii* PP664439 (MG 3),‏ h: normal ovoid cell, i-‏j‏: ‏EO1 –PDA i, apoptotic cell with condensed chromatin, destroyed wall and plasma membrane, leakage,‏ j, early ‏apoptotic cells with ‏collapsed cytoplasm. k-l: EO2 –PDA, k; late apoptotic cell, leaked cytoplasm (L), degrade wall at bud scar site (B), collapsed cytoplasm, l, necrotic cell empty from cytoplasm, leaked cytoplasm (L), degrade wall at bud scar site (B). Bars in ‏µm as illustrated on each image.

The transmission electron microscopy (TEM) analysis provided detailed evidence of the antifungal activity of EO1-PDA and EO2-PDA nano-capsules against *C. albicans* PP664305 (MG1), *P. kudriavzevii* PP664417 (MG2), and *P. kudriavzevii* PP664439 (MG3). Untreated fungal cells exhibited an intact morphology with a well-defined cell wall (CW), plasma membrane (CM), cytoplasm full of electron-dense chromatin material (C), and prominent nucleus (N), indicating their normal physiological state (Fig. 8a, e, h). The cell walls of these fungi measured approximately 103 nm in thickness, appearing structurally stable without any signs of damage. Upon treatment with EO1-PDA and EO2-PDA nano-capsules, significant cellular damage was observed, with notable differences in the type and extent of disruption. EO1-PDA treatment primarily induced early apoptotic changes, including chromatin condensation, irregular plasma membrane morphology, and apoptotic cell bursting, as seen in *C. albicans* (Fig. 8b), *P. kudriavzevii* PP664417 (Fig. 8f), and *P. kudriavzevii* PP664439 (Fig. 8i). The plasma membrane became irregular, and intracellular contents started condensing, suggesting an early-stage apoptotic response. In some cases, cytoplasmic leakage and partial wall degradation were also visible (Fig. 8d), indicating progressive cellular collapse. In contrast, EO2-PDA treatment caused more severe damage, leading to late apoptosis and necrosis. Cells exhibited complete cytoplasmic disintegration, nuclear material leakage, and extensive wall degradation. For instance, Fig. 8c (*C. albicans*) and Fig. 8l (*P. kudriavzevii* PP664439) show necrotic cells completely devoid of cytoplasmic content, suggesting cell death through membrane rupture and organelle disintegration. Similarly, Fig. 8k highlights an apoptotic *P. kudriavzevii* PP664439 cell with collapsed cytoplasm, a degraded wall at the bud scar site (B), and extensive leakage (L), confirming that EO2-PDA was more potent in inducing irreversible cellular damage. Comparing the effects across the three fungal strains, *C. albicans* (MG1) appeared highly susceptible, showing rapid apoptotic changes and cell bursting (Figs. 8b and c), whereas *P. kudriavzevii* (MG2 and MG3) displayed a mix of early and late apoptotic features, with some cells progressing to necrosis under EO2-PDA treatment (Figs. 8f, g, i and j). The differences in structural collapse may be attributed to species-specific variations in cell wall composition and membrane integrity^83,84^. Overall, these TEM findings confirm that EO-based nano-capsules effectively disrupt fungal cell integrity through apoptotic and necrotic pathways, with EO2-PDA exhibiting stronger cytotoxic effects. The observed cellular damage ranging from chromatin condensation and membrane irregularities to complete cytoplasmic leakage and wall degradation highlights the potential of these nano-capsules as potent antifungal agents. Their ability to induce programmed cell death (apoptosis) and necrosis suggests a dual mechanism of action, making them promising candidates for combating resistant fungal pathogens.

## Conclusion

This study demonstrates that encapsulating *Cupressus macrocarpa* and *Mentha longifolia* essential oils in polydopamine nanocapsules significantly enhances their antifungal activity against *Candida albicans* and *Pichia kudriavzevii*. The EO-PDA formulations achieved lower MIC values compared to the free oils, and TEM analysis revealed characteristic signs of fungal cell damage, including membrane disruption and cytoplasmic leakage. The nanocapsules also exhibited dose-dependent cytotoxic activity against HepG-2 and MCF-7 cancer cell lines and demonstrated antioxidant potential. These findings support the potential of EO-loaded PDA nanocapsules as a promising platform for antifungal applications, particularly where enhanced efficacy at reduced doses is desirable. Overall, the complementary antioxidant and antiproliferative activities of the EO–PDA nanocapsules reinforce their antifungal efficacy, highlighting their potential as multifunctional and biocompatible therapeutic systems. Given these findings, EO-PDA nanocapsules represent a novel, biocompatible, and effective strategy for managing oral fungal infections and related diseases. Further studies are warranted to explore long-term stability and in vivo safety.

## Supplementary Information

Below is the link to the electronic supplementary material.

Supplementary Material 1

## Data Availability

The datasets used and/or analyzed during the current study available from the corresponding author on reasonable request.
