# Supplementary material for: Enhanced antifungal and cytotoxic potential of essential oils encapsulated in polydopamine nanocapsules against Candida albicans and Pichia kudriavzevii
Source: Sci Rep. 2026 Mar 11;16:8955. doi: 10.1038/s41598-026-40233-y (PMC12988115; doi:10.1038/s41598-026-40233-y)
Supplement: Supplementary file 1 — Supplementary Material 1 [file 41598_2026_40233_MOESM1_ESM.docx]

**Supplementary information for**

**Enhanced Antifungal and Cytotoxic Potential of Essential Oils Encapsulated in Polydopamine Nanocapsules Against *Candida albicans* and *Pichia kudriavzevii***

El-Sayed M. El-Morsy ^a^, Marwa T Mohesien ^a^, , Mohamed Alghzaly Mohamed Abdellatif ^a^, Elsayed Elbayoumy ^b^

*^a^ Microbiology and Botany Department, Faculty of Science, Damietta University, New Damietta 34517, Egypt.*

*^b^ Chemistry Department, Faculty of Science, Damietta University, New Damietta 34517, Egypt.*


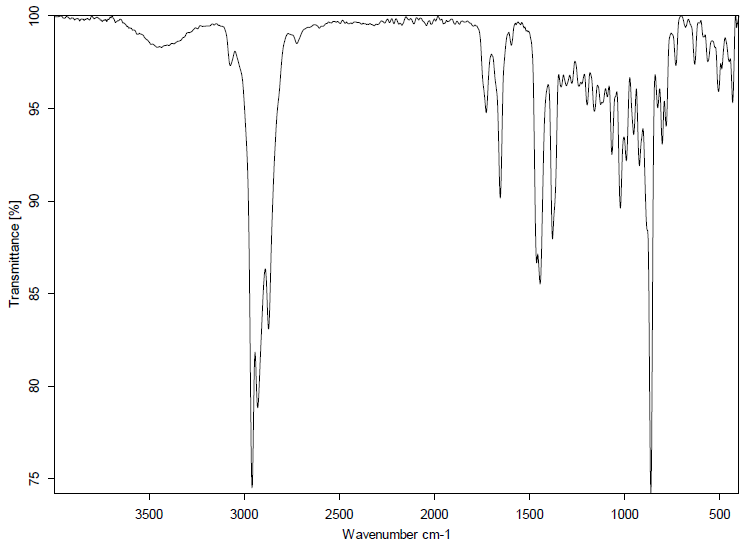


Figure S 1. FTIR spectra of EO1


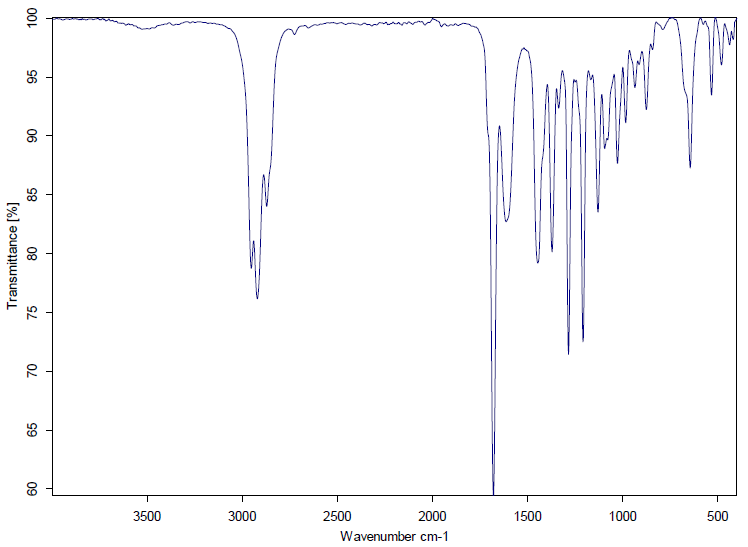


Figure S 2. FTIR spectra of EO2.

Figure S 3. Calibration curve for determining the amount of EO1 (A) and EO2 (B).

Figure S 4.TEM particle size distribution of EO1-PDA (A) and EO2-PDA (B) capsules.

Figure S 5. Evaluation of cytotoxicity against HepG-2 cell line and MCF-7 cell line for (A) free EO1 and (B) free EO2.

Figure S 6. Evaluation of cytotoxicity against HepG-2 cell line and MCF-7 cell line for PDA

v

Figure S 7. Evaluation of Antioxidant Activity using DPPH scavenging of free EO1, free EO2 and PDA
